# Assessment of Ethnic Inequities and Subpopulation Estimates in COVID-19 Vaccination in New Zealand

**DOI:** 10.1001/jamanetworkopen.2022.17653

**Published:** 2022-06-21

**Authors:** Andrew Anglemyer, Corina Grey, Collin Tukuitonga, Andrew Sporle, Gerard J. B. Sonder

**Affiliations:** 1 Department of Preventive and Social Medicine, University of Otago, Dunedin, New Zealand; 2 Health Intelligence Team, Institute of Environmental Science and Research, Wellington, New Zealand; 3 Vaka Tautua, Auckland District Health Board, Auckland, New Zealand; 4 Faculty of Medical and Health Sciences, University of Auckland, Auckland, New Zealand; 5 Statistics Department, University of Auckland, Auckland, New Zealand; 6 Department of Internal Medicine, Amsterdam Infection and Immunity Institute, Amsterdam UMC, University of Amsterdam, Amsterdam, the Netherlands

## Abstract

This cross-sectional study investigates the outcomes of different population estimate methodologies on relative gaps in COVID-19 vaccination between ethnic groups and the resulting population risk among people in New Zealand.

## Introduction

COVID-19 has exposed inequities in access to care, baseline health, and economic standing.^1,2^ In Aotearoa New Zealand, Pacific peoples and Māori have disproportionately experienced poor SARS-CoV-2 outcomes. To ensure prevention and care equity, the COVID-19 vaccination program targeted high-risk people first. In October 2021, the Ministry of Health (MoH) announced 90% vaccine coverage targets among eligible populations, obviating the need for future lockdowns.^3^ Beginning December 2021, vaccination proof was required for everyone aged at least 12 years to access certain venues (eg, hospitality services); Pfizer-BioNTech (BNT162b2) booster vaccines were required for specific occupations (eg, health care). We highlight the outcome of different population estimate methodologies on relative gaps in vaccination between ethnic groups and the resulting population risk.

## Methods

This cross-sectional study followed the Strengthening the Reporting of Observational Studies in Epidemiology (STROBE) reporting guideline. This study is ethics review exempt and did not require informed patient consent because it includes only public data with no sensitive health data, in accordance with section 4 part 134ii of the New Zealand Ministry of Health’s Operational Standard for Ethics Committees.

We collated publicly available MoH vaccination data^4^ using Health Service Utilization (HSU) data to estimate subpopulation sizes^4^ (including individuals who used health services in 2020 and used to determine vaccination targets). For comparison, we used Stats NZ 2020 Estimated Resident Population data for subpopulation sizes of ethnic groups, which were reported in health records and include European/other, Māori, and Pacific peoples, using the standard MoH ethnicity prioritization method. We defined broad age- and vaccination-based risk classifications as follows: (1) among individuals vaccinated with 2 doses (ie, some protection from symptomatic disease), severe illness risk increases with age, although low (absent comorbidities) until 70 years of age,^5^ (2) among unvaccinated individuals, severe illness also increases with age, although risks are moderate from 50 years of age and high from 65 years of age. No confidence intervals were constructed as the uncertainty in estimates stems from denominators required for the estimator, not from sampling variability. χ^2^ tests were used with a 2-sided *P* < .05 significance threshold. Statistical analysis was performed using R version 4.03 from March to April 2022.

## Results

**Figure 1.**
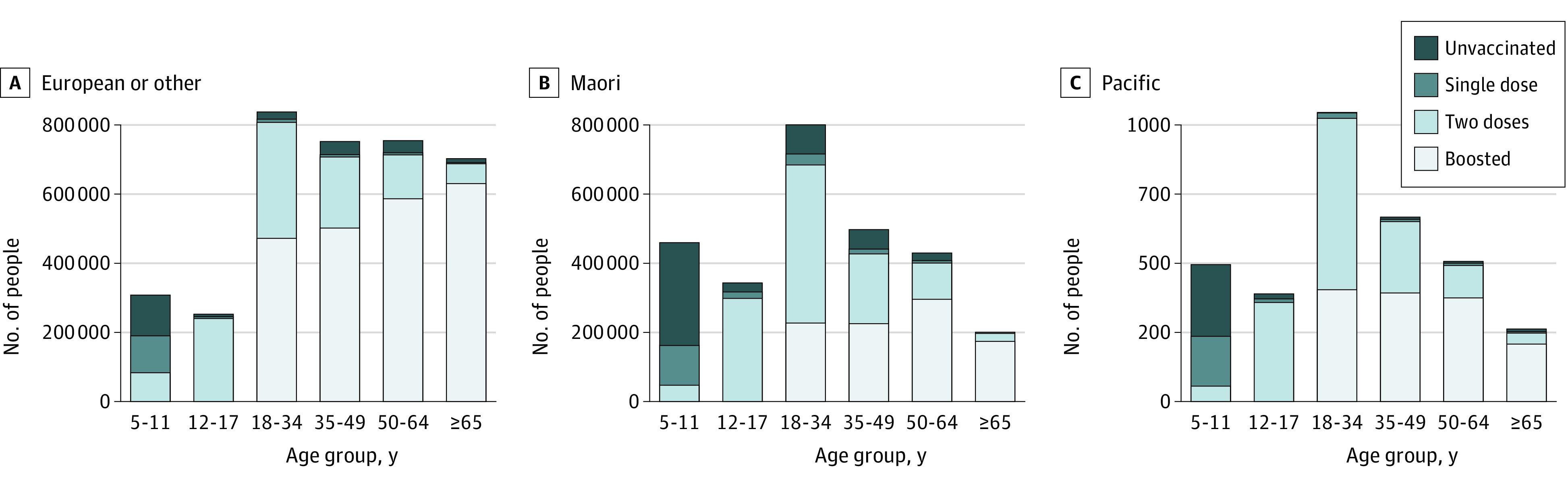
COVID-19 Vaccine and Booster Coverage by Ethnicity and Age Group

The proportion of Māori individuals aged at least 12 years who were fully vaccinated (88.2%; 503 718 of 571 052) was significantly less than individuals identifying as European/other (95.6%; 3 170 979 of 3 318 148) and Pacific peoples (96.5%; 276 596 of 286 681) (*P* < .001). There were early gaps in the vaccine rollout for children aged 5 to 11 years, with 57% (128 377 of 223 785) of European/other children vaccinated (at least 1 dose), followed by 47% (23 335 of 49 398) of Pacific children and only 35% (40 658 of 115 562) of Māori children (Figures 1A, 1B, and 1C). Similarly, significantly higher proportions of eligible European/other individuals (71.9%; 2 202 299 of 3 063 530) have been boosted compared with Māori (47.9%; 232 367 of 484 804) and Pacific (55.8%;138 290 of 247 670) individuals (*P* < .001).

**Figure 2.**
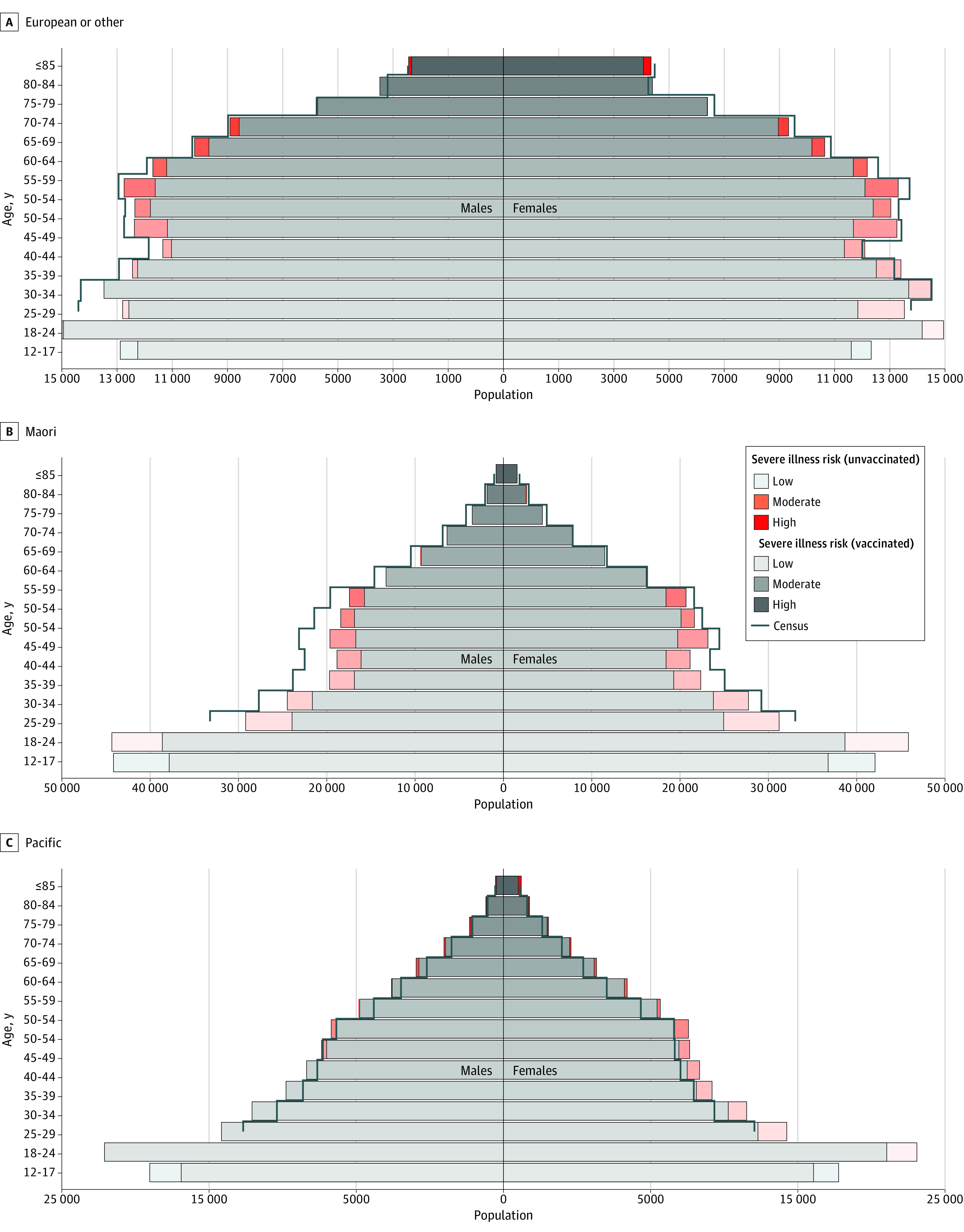
Population Pyramids by Ethnicity, COVID-19 Vaccine Coverage, and Eligible Subpopulation Sizes Using HSU and Census Estimates Everyone aged at least 5 years was eligible for vaccination, although vaccination data stratifications by ethnicity, age, and gender are only available for those at least 12 years of age. Census data with equivalent granularity of vaccination data stratifications are only available for people aged at least 25 years.

The population pyramids in Figure 2 show not only different population distributions between ethnic groups, but highlight how different population estimates can impact vaccine coverage estimates. Using HSU subpopulation estimates, 3.5% (51 171 of 1 465 677) of European/other individuals, 2.7% (2085 of 76 672) of Pacific peoples, and 3.2% (5029 of 157 125) of Māori individuals who were aged 50 years or older were classified as moderate or high risk due to their age and unvaccinated status. Overall, there were approximately 718 948 unvaccinated residents, of whom 76% (543 211) were younger than 12 years of age. If census data (represented by black outlines in Figures 2A, 2B, and 2C) in lieu of HSU data were used, the proportion of vaccinated female Pacific peoples (aged at least 25 years) would increase 6.3% (no difference for male Pacific peoples). However, the proportion of vaccinated Māori individuals (aged at least 25 years) would decrease 5.0% for female individuals and 12.0% for male individuals.

## Discussion

In this study, the choice of denominator affected coverage estimates disproportionately between ethnic groups. Because HSU only captures people who use health services, younger and marginalized populations are likely underrepresented, leading to overestimates of coverage in these groups. However, if we use census data we may underestimate subpopulation sizes of Pacific peoples,^6^ which could lead to underestimates of unvaccinated, highlighting the difficulties in using census data to estimate accurate subpopulation counts in dynamic, migrant populations. Although true disparities in vaccinations are unclear, ethnic minority groups should continue to be reached through targeted engagement. Without accurate census estimates of subpopulations aged under 25 years, our comparisons between HSU and census data are limited.
