# Errors in Figure

**DOI:** 10.1001/jamanetworkopen.2022.26752

**Published:** 2022-07-22

In the Research Letter titled “Assessment of Ethnic Inequities and Subpopulation Estimates in COVID-19 Vaccination in New Zealand,”^1^ published June 21, 2022, the y-axis scale was incorrect in Figure 1B and 1C. The y-axis should have spanned from 0 to 200 000 in Figure 1B, and from 0 to 100 000 in Figure 1C. This article has been corrected.^1^
